# Induction and Marker Selection of Embryogenic-like Callus from the Hypocotyl of *Pinus thunbergii*

**DOI:** 10.3390/plants15142140

**Published:** 2026-07-10

**Authors:** Jing Dai, Lijuan Gao, Mengyu Zhang, Jing Liu, Peng Meng

**Affiliations:** 1 School of Landscape Architecture and Forestry, Qingdao Agricultural University, Qingdao 266109, China; 15263116578@163.com (J.D.); m17860721038@163.com (L.G.); 18764462331@163.com (M.Z.); liujing01680@163.com (J.L.); 2 Shandong Key Laboratory for Germplasm Innovation of Saline-Alkaline Tolerant Grasses and Trees, Qingdao 266109, China; 3 Leshan Engineering Technology Research Center for Innovative Utilization of Feature Plant Resources, Leshan 614000, China

**Keywords:** *Pinus thunbergii*, hypocotyl, embryogenic-like callus, peroxidase, proteomics

## Abstract

To induce embryogenic callus from *Pinus thunbergii* vegetative organs, and deeply understand the internal mechanism of the process, an orthogonal experimental design of three factors and four levels and proteome analysis were adopted. The results showed that the optimal medium was douglas-fir cotyledon medium (DCR), containing 2.5 mg L^−1^ 2-4-dichlorophenoxyacetic acid (2,4-D) and 1.0 mg L^−1^ 6-benzylaminopurine (6-BA), 0.3 g L^−1^ polyvinylpyrrolidone (PVP), 0.5 g L^−1^ acid casein hydrolysate (CH), 0.5 g L^−1^ L-glutamine, 1.0 g L^−1^ inositol, 6.0 g L^−1^ agar and 20.0 g L^−1^ sucrose. Microscopic examinations revealed a distinct embryogenic-like callus (EC) structure, and these ECs finally achieved redifferentiation. Analysis of the interactions between factors detected that although the 6-BA concentration alone was not significant, it became a significant effect factor when interacted with 2,4-D (*p* < 0.05). Peroxidase (POD), superoxide dismutase (SOD) and soluble sugar (SS) of EC were significantly higher than those of non-embryogenic callus (NEC), and label-free quantitative proteomics analysis showed that different types of PODs including peroxidase 4, phospholipid hydroperoxide glutathione peroxidase, and cationic peroxidase 1 in EC were significantly up-regulated, and they were involved in antioxidant biological processes, located in the intercellular region, and performed molecular functions such as heme binding, so POD was a suitable and stable physiological marker for EC. Four up-regulated proteins in EC included glutathione S-transferase, chalcone flavanone isomerase, phosphoenolpyruvate carboxykinase and endoglucanase. Five EC-specific proteins included indole-3-acetic acid-amido synthetase GH3.1, indole-3-acetate O-methyltransferase 1-like, cytokinin dehydrogenase, MLP-like protein 423 and 2-methoxy-6-polyprenyl-1,4-benzoquinol methylase. These proteins are also potential EC molecular markers. Among these proteins, glutathione S-transferase is beneficial to prevent cell death in EC, while indole-3-acetate O-methyltransferase 1-like and cytokinin dehydrogenase are beneficial to promote EC redifferentiation.

## 1. Introduction

*Pinus thunbergii* is a famous species used for the greening of coastal areas as well as afforestation of barren mountains and landscaping in China. However, the emergence of pine wilt disease in some areas has led to the loss of landscape value of *P. thunbergii*, and then some genotypes resistant to pine wood nematode have been found. It is of great significance to propagate these excellent genotypes. Seed propagation is currently the main method used for propagating *P. thunbergii*, but a disadvantage of this method is the variation or loss of excellent parental traits. Somatic embryogenesis is applicable for mass reproduction, and it may also contribute to the development of biotechnology-based methods for generating new forestry products [1]. Somatic embryogenesis of *P. thunbergii* usually used immature zygotic embryos as explants [2], while there are no reports regarding the use of hypocotyls or needles as explants.

Propagation using vegetative organs can better maintain the excellent traits of the parent species, and the period of sampling is broader. Somatic embryogenesis using the vegetative organs (EVO) generally involves two methods, namely direct organogenesis and indirect organogenesis [3]. Pinaceae species generally adopt indirect organogenesis. Ran et al. used *P. massoniana* apical buds as explants and induced callus in DCR medium supplemented with 2,4-D, naphthaleneacetic acid (NAA), and 6-BA, and compared the differentially expressed genes of callus under different additives by transcriptome sequencing, but did not discuss the embryogenicity of callus [4]. Another study demonstrated that the slight browning of some induced *P. virginiana* ECs after an incubation period inhibits the subsequent proliferation [5]. These earlier studies indicated that EVO of Pinaceae plants is challenging, with most of the induced calli revealed as non-embryogenic and difficult to proliferate further. Therefore, understanding the molecular mechanism of somatic cell dedifferentiation and regeneration in the EVO process of pine tree species is crucial for optimizing in vitro regeneration.

Although the EVO pathway was rarely reported in pine conifers, this technique has been recently reported in some broad-leaved woody plants such as pomegranate (*Punica granatum*, using stem explants) [6], honeysuckle (*Lonicera caerulea*, using cotyledon explants) [7], and tree tomato (*Solanum betaceum*, using leaf explants) [8]. EVO involves four steps [9], and the induction of ECs from vegetative organs is the first step, but research in this field for pine conifers has been limited. In this step, basic medium types and supplemented organic matters (e.g., PVP, CH, L-glutamine and inositol) may have important effects, and the types and concentrations of exogenous auxin and cytokinin were also critical factors [10,11]. The synthetic auxin 2,4-D was chemically more stable and 10- to 1000-times more active than natural auxins (e.g., indole-3-acetic acid, IAA) [12], and was often involved in the EC induction [13]. Some reactive oxygen species (ROS) were toxic to cells and inhibited the formation of embryogenic callus [14]. Peroxidase (POD) and superoxide dismutase (SOD) in plants can prevent and alleviate the damage of excessive accumulation of ROS and other peroxide free radicals to the cell system [15]. Soluble sugar (SS) would provide energy to metabolically active embryonic cells [16]. Many enzymes or transcription factors directly affected the biochemistry of cells, so proteomics was usually used to analyze zygotic and somatic embryogenesis development [17,18]. In previous studies, proteomic techniques were used to analyze the differences between EC and NEC of *Larix principis-rupprechtii* [19] and *P. nigra* [20]. Three EC-specific proteins were found in *L. principis-rupprechtii*, and two EC-specific proteins were found in *P. nigra*. These proteins or sugar may also provide physiological and molecular markers for EC used to classify the early cultured calli.

According to the above results, in the current study, four basic media differing greatly in terms of their nitrogen content and different ratios of ammonium nitrogen (NH_4_^+^) and nitrate nitrogen (NO_3_^−^), supplemented with four concentrations of 6-BA and 2,4-D, were used to induce calli; then, the induced calli were examined regarding their morphological and cytological characteristics, as well as POD activity, SOD activity, SS content and label-free quantitative proteomics. The goal of our study was to investigate the effects of the basic media and the different 2,4-D and 6-BA concentrations on the induction of callus from hypocotyls by performing orthogonal tests, deeply understand the mechanism of induction and redifferentiation of EC from the aspects of morphology, physiology and protein, and provide researchers with technical insights relevant to the propagation of high-quality *P. thunbergii* by EVO.

## 2. Results and Analysis

### 2.1. Morphology and Cytology of Callus

**Figure 1:**
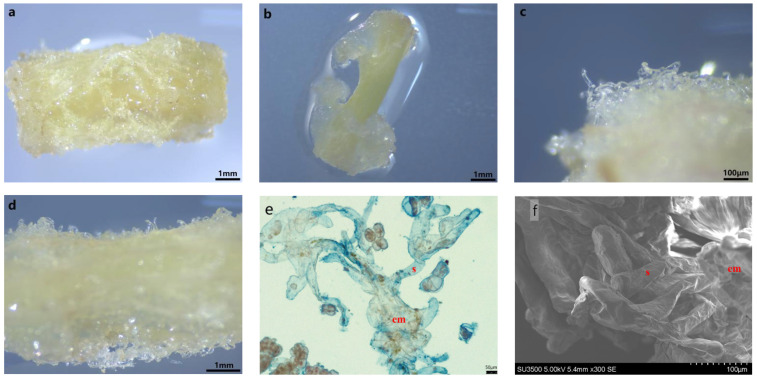

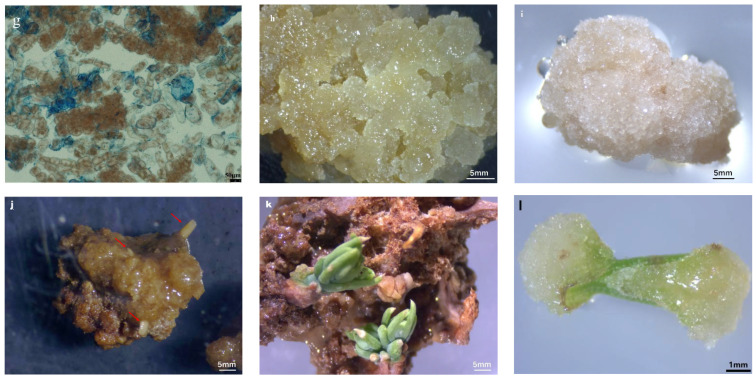
Observation of calli induced from hypocotyls (**a**–**k**) and needles (**l**) ((**a**,**b**): Callus of different growth states at the induction stage. (**c**,**d**): Observation of embryonic cells under stereoscopic microscope. (**e**,**f**): The callus with EC structure characteristics was observed under biological microscope after staining and stereoscopic microscope after two subcultures (em—embryogenic head, s—suspensor cell). (**g**): The NEC was observed under biological microscope after staining. (**h**,**i**): The surface structure of EC and NEC after subculture for 4 times was observed under stereoscopic microscope. (**j**): Differentiation of somatic embryos (shown in red arrow). (**k**): Differentiation of plantlet. (**l**): Callus induced from one-year needles.)

After the hypocotyl explants were cultured on the induction medium for 7 days, white callus began to form at both ends of the incision. With the prolongation of culture time, the number and size of the callus gradually increased, as well as the overall expansion and cracking of the explant segments (Figure 1a) or the formation of a dumbbell shape (Figure 1b). The existence of EC could be observed under the stereomicroscope (Figure 1c,d). The callus was observed under a biological microscope, and several vacuolated suspensor cells were observed in the embryogenic head (Figure 1e), which is characteristic of EC [21]. Under a scanning electron microscope, the suspensor cells were clearly visible (Figure 1f), as well as the other type of mostly round cells with darker granular color (Figure 1g), which were NECs. After four subcultures, the surface of EC appeared to have more layers and protrusions, while NEC was relatively smooth and had few protrusions (Figure 1h,i). About 180 days later, EC differentiated into somatic embryos, and differentiated into plantlets after two months (Figure 1j,k).

### 2.2. Selection of the Best Combination for EC Induction

The EC induction rates from hypocotyls among the 16 protocols were compared and extreme difference (R) analysis was performed (Table 1). It can be seen that the order of the magnitude was R_B_ > R_C_ > R_A_, which meant the order of factor effects was 2,4-D concentration > 6-BA concentration > medium type, and A_4_B_4_C_2_ was the optimal combination for this experiment based on the k-value, that was DCR + 2.5 mg L^−1^ 2,4-D + 1.0 mg L^−1^ 6-BA.

As shown in Table 2, the R^2^ value of the ANOVA was 0.944, indicating that these three factors could explain 94.4% of the variation in the data. According to the *p*-value, only the 2,4-D concentration had a significant effect on callus induction rate (*p* < 0.01). There were no significant effects of interaction between the medium type and 2,4-D concentration or between the medium type and 6-BA concentration on the EC induction rate. But for the B × C interaction, F_B×C_ was 47.285, far greater than F_crit_, indicating that the interaction between 2,4-D concentration and 6-BA concentration had a significant effect on the callus induction rate. The concentration level of 6-BA by itself had no significant effect on the experimental results, but when it was combined with 2,4-D there was a significant interaction.

**Figure 2:**
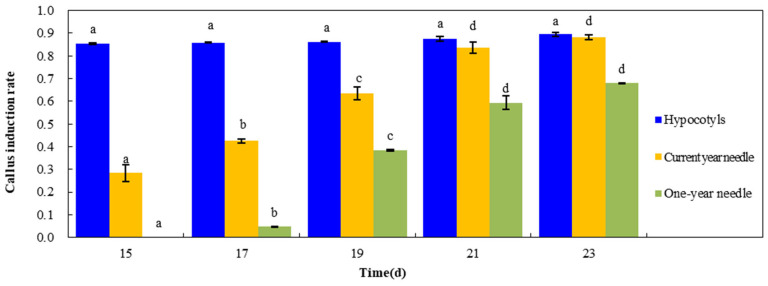
The change in callus induction rate from hypocotyls and needles with time (each treatment was examined with 120 biological replicates. The media used in each treatment was the selected combination according to the above orthogonal experiment. Different lowercase letters indicated that there were significant differences in the same explant under different culture time treatments.)

The results of the post hoc multiple comparison showed that there were significant differences between 2,4-D concentration levels. The average EC induction rate of 1.5, 2.0, and 2.5 mg L^−1^ levels was significantly higher than that of the 1.0 mg L^−1^ level (*p* < 0.01, Table 3). Using the best combination above, another callus induction test adopting current year and one-year needles of *P. thunbergii* as explants was also carried out, and the callus started to appear around 15 d (Figure 1l). However, the overall rate of callus production in needles was slower than that in hypocotyls (Figure 2), and those calli were NEC.

### 2.3. Analysis of Antioxidant Enzyme Activity and SS Content Between EC and NEC

POD, SOD and SS were determined by using EC and NEC from hypocotyls as samples. The results in Table 4 showed that the activities of POD and SOD in EC were significantly higher than those in NEC (*p* < 0.05), and the former were 1.64 and 2.00 times of the latter, respectively, although there was no significant difference in CAT between the two. In the two samples, the SS content of EC was also significantly higher than that of NEC (*p* < 0.01), and the former was even 2.23 times that of the latter.

### 2.4. Proteomics Analysis Between EC and NEC

**Figure 3:**
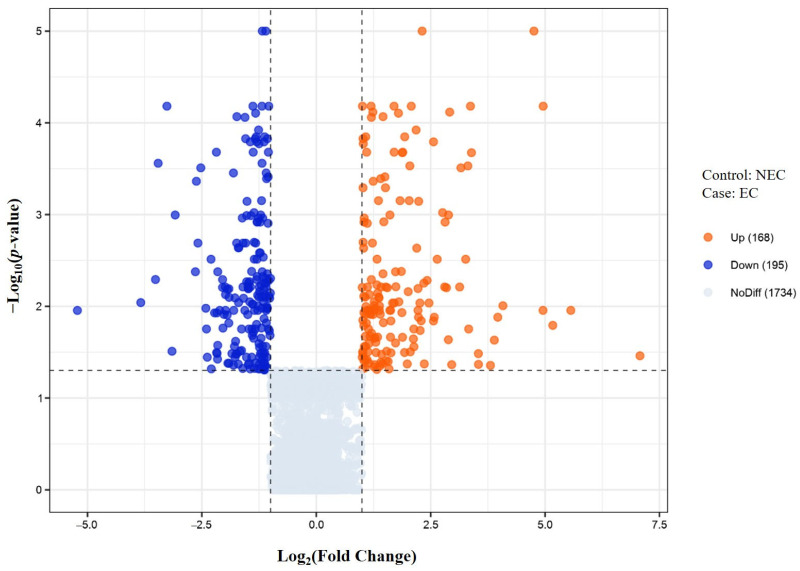
Volcano map of proteins (two vertical dashed lines in the figure are 2 times the threshold of differential expression, and the horizontal dotted line is the *p*-value = 0.05 threshold. Red dots represent up-regulated DEPs, blue dots represent down-regulated DEPs, and gray dots represent non-significant differentially expressed proteins.)

**Figure 4:**
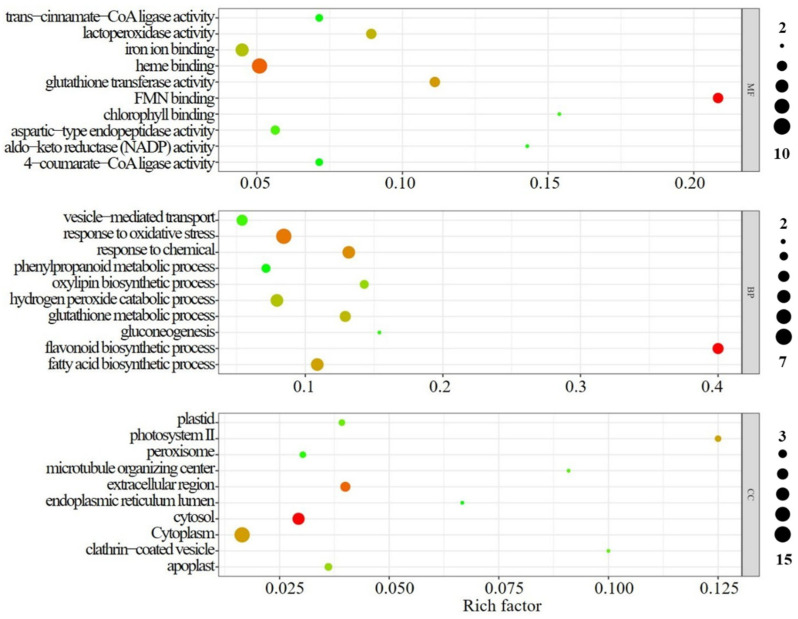
GO enrichment analysis of up-regulated DEPs (the color of the bubble represents the significance of the enrichment, that is, the *p*-value calculated based on Fisher’s Exact Test, the color gradient represents the size of the *p*-value, and the color gradually changes from green to red. The closer to red, the smaller the *p*-value, and the higher the corresponding significance level. The value of pl in the panel is −log_10_p. The size of the bubble represents the number of DEPs.)

A total of 3215 proteins were detected, of which 363 proteins were DEPs (Figure 3), and 628 proteins were specifically expressed in EC. GO enrichment analysis for those up-regulated DEPs was performed, including three categories: molecular function (MF), biological process (BP) and cellular component (CC) (Figure 4). The presence of PODs was detected in all three types of significantly up-regulated differential proteins (Table 5), such as antioxidant response in biological process (including peroxidase 4, phospholipid hydroperoxide glutathione peroxidase, cationic peroxidase 1), heme binding in molecular function (including peroxidase 4 and cationic peroxidase 1), and extracellular regions in cellular components (including peroxidase 4 and cationic peroxidase 1). In addition, the SOD expression gene of EC in cell components was also significantly up-regulated, but only in the cytoplasm.

As shown in Figure 4 and Table 5, in terms of MF, the DEPs significantly up-regulated in EC were concentrated in FMN (Flavin Mononucleotide) binding, heme binding and glutathione transferase activity. Among them, the number of DEPs in heme binding function was as high as 11 proteins, while in the FMN binding function, although the number of DEPs was not large (five proteins), its enrichment factor was the highest, at about 0.208. In terms of BP, the DEPs significantly up-regulated in EC were concentrated in response to oxidative stress, flavonoid biosynthetic processes and responses to chemicals. Among them, the response to the oxidative stress process had the largest number of proteins (seven proteins), while the flavonoid biosynthetic process had the highest enrichment factor (0.4). The protein with the highest expression level in this process was chalcone flavanone isomerase (EC 5.5.1.6). In terms of CC, the significantly up-regulated proteins in EC were widely distributed in cytosol, the extracellular region, and the cytoplasm, with the numbers of 10, six, and 18 proteins, respectively. The highly expressed proteins included phosphoenolpyruvate carboxykinase (EC 4.1.1.32) and endoglucanase (EC 3.2.1.4).

This study also found several types of proteins specifically expressed in EC: 1. Mitosis and cell cycle-related proteins, such as sister chromatid cohesion protein PDS5 homolog A (gene id 9268318, TRINITY_DN8494_c0_g1.p1), and cell division control protein 48 homolog E (gene id 4348731, TRINITY_DN21027_c0_g1.p1). 2. Auxin-related proteins, such as probable indole-3-acetic acid-amido synthetase GH3.1 (gene id 4327043, TRINITY_DN1290_c0_g1.p1), indole-3-acetate O-methyltransferase 1-like (gene id 4337315, TRINITY_DN16252_c0_g1.p1), and tetraspanin-8 (gene id 4347111, TRINITY_DN1980_c0_g1.p1). 3. Cytokinin-related proteins, such as cytokinin dehydrogenase (gene id 4345764, TRINITY_DN3614_c0_g1.p1). 4. Abscisic acid-related proteins, such as MLP-like protein 423 (gene id 4336091, TRINITY_DN21357_c0_g1.p1, TRINITY_DN9839_c0_g1.p1). 5. Ubiquinone synthesis-related proteins, such as 2-methoxy-6-polyprenyl-1,4-benzoquinol methylase (gene id 4324310, TRINITY_DN42870_c0_g1.p1).

## 3. Discussion

### 3.1. Optimal Culture Media and Hormones for EC Induction

This study preliminarily explored the feasibility of EVO technology on *P. thunbergii*, and found the feasible explants were hypocotyls, and selected the best EC induction combination through an orthogonal experiment. The results showed that the best medium was DCR, which may be related to the lower contents of NO_3_^−^ and NH_4_^+^ and the well-balanced ratio between them. DCR was reported to be the most suitable medium for EC induction in *P. bungeana*, *P. taeda* and *Larix kaempferi* [22,23]. The necessity of plant hormone for somatic embryogenesis has been confirmed in a variety of plants [24]. This study showed that 2,4-D had a significant effect on the induction of hypocotyl callus of *P. thunbergii*. When the concentration of 2,4-D was 2.5 mg L^−1^, the induction rate reached the maximum. It has been reported that explants cultured in a medium containing 2,4-D changed the endogenous auxin level of the corresponding explants, which was one of the key signals to determine embryogenesis [25,26]. 2,4-D might act as a stressor that triggers embryonic development in cultured plant cells [27], thereby promoting the transformation of somatic cells to embryonic cells. In *Larix olgensis*, the EC induction rate increased with 2,4-D concentration within a certain range [28]. The interaction analysis of orthogonal test in this study also showed that there was a significant interaction between 2,4-D and 6-BA, indicating that 6-BA would enhance the effect of 2,4-D, and the combination of them was more conducive to EC induction. Some studies also found that the EC induction rate of 2,4-D alone was significantly lower than that of 2,4-D and 6-BA [12]. In addition, the organic additives used in this study, such as glutamine, CH, PVP, etc., also promoted the formation of EC, which was also reported in other studies [29]. Although the induction rate of EC in this study is high, the proportion of EC that can form somatic embryos is still low, only about 20%. In addition, the number of somatic embryos formed by each EC is 3–5, which is also relatively small. This reflects the difficulty of the EVO pathway and is also the focus of future research.

### 3.2. Optimal Physiological Marker for Early Identification of EC

Previous studies have shown that the high content of reactive oxygen species (ROS) in NEC is an important factor leading to programmed cell death (PCD) and loss of embryonicity [30]. In our study, the activity of EC antioxidant enzyme was higher, which effectively reduced the content of ROS in cells, thus inhibiting cell death. Our study found that POD content in EC was significantly higher than that in NEC. Proteomics analysis also showed that the up-regulated expression of PODs in EC, and those PODs including peroxidase 4, phospholipid hydroperoxide glutathione peroxidase, and cationic peroxidase 1. Peroxidase 4 belongs to the class III secretory plant peroxidase, which is involved in a variety of biochemical processes, including the synthesis of cell wall components, hormone regulation and defense mechanisms. Phospholipid hydroperoxide glutathione peroxidase is a special kind of peroxidase, which plays a central role in cellular antioxidant defense and ferroptosis regulation. Cationic peroxidase 1 is widely present in plants and is involved in plant defense responses, lignin biosynthesis and ROS metabolism [31]. Our results showed that these PODs in EC were involved in antioxidant biological processes, located in the intercellular region, and performed molecular functions such as heme binding. Since multiple types of POD are up-regulated, this index could be used as an effective and stable marker for early identification of EC. It has been reported that POD played a major role in the somatic embryo induction stage and promotes somatic embryogenesis in larch [32]. The mechanism of POD activity promoting somatic embryogenesis might be that the EC cells maintain strong differentiation ability, material metabolism and respiration during early somatic embryogenesis [33]. In our study, SOD activity in EC is also higher than that in NEC, and this result is consistent with Liu et al. in the study of somatic embryogenesis of alfalfa [34]. However, from the proteome determination, SOD was only located in the cytoplasm and was not significantly involved in the antioxidant process, so compared with POD, SOD was not a stable indicator of embryonicity. Our study also found that the SS content of *P. thunbergii* EC was significantly higher than that of NEC, indicating that the metabolic rate of EC was higher, and the high concentration of SS provided energy for the EC production.

### 3.3. Potential Molecular Marker for Early Identification of EC

In terms of MF, there was a significant difference in heme binding between EC and NEC, and the expression of heme binding-related proteins was up-regulated in EC (Figure 4). Some studies found that the expression of heme activator protein was significantly up-regulated in EC, and its ectopic expression was beneficial to induce somatic embryogenesis of vegetative cells [35]. The high expression of FMN binding function meant that the EC respiratory chain had high redox activity. This study also found that glutathione S-transferase (EC 2.5.1.18) was highly expressed in EC, and the expression of this protein was beneficial to callus morphogenesis [36].

In terms of BP, the flavonoid biosynthetic process had the highest enrichment factor, and the most typical protein was chalcone flavanone isomerase, which was a key enzyme in the flavonoid biosynthesis pathway. Studies have shown that flavonoids are involved in the early somatic embryogenesis of various plants [37]. In addition, glutathione S-transferase, which was highly expressed in EC, was also involved in the transport of flavonoids from the cytoplasm to vacuole [38]. Studies have shown that PCD often occurs in NEC [39], and the presence of high concentrations of flavonoids in the cytoplasm can cause toxicity to cells and even promote cell death. The high expression of glutathione S-transferase in EC can transfer flavonoids from the cytoplasm to the vacuole, which not only reduces the risk of cell death, but also enables the accumulation of flavonoids in the vacuole to better exert their antioxidant and defensive effects.

In terms of CC, the expression of phosphoenolpyruvate carboxykinase in cytosol was high, and this enzyme was one of the key enzymes of gluconeogenesis and was beneficial to promote the conversion of non-sugar substances to sugars in plants, which was also the reason for the high SS in EC (Table 4). In the extracellular region, endoglucanase had the highest expression level. The role of endoglucanase in somatic embryogenesis was mainly reflected in the degradation of the cell wall and the regulation of embryonic callus development. It may promote the expansion of embryonic cells by degrading cell wall components (such as β-1,3-glucan) [40].

Among the EC-specific proteins, indole-3-acetic acid-amido synthetase GH3.1 can catalyze the binding of plant hormones such as auxin (IAA), jasmonic acid (JA) and salicylic acid (SA) to amino acids, regulate the concentration of plant hormones, and regulate plant growth and development and stress response processes [41]. The protein can also regulate the signal transduction of assembly reaction factor (ARF) protein [42]. Indole-3-acetate O-methyltransferase 1-like is involved in maintaining auxin homeostasis, while tetraspanin-8 is widely involved in auxin signal transduction pathways. Cytokinin dehydrogenase is responsible for maintaining the homeostasis of endogenous cytokinin. The formation process of the meristem is the coordination process of endogenous auxin and the meristem, and the two interact with each other [43]. Indole-3-acetate O-methyltransferase 1-like and cytokinin dehydrogenase are involved in maintaining the homeostasis of auxin and cytokinin, which is conducive to the formation of the meristem (e.g., proembryo). MLP-like protein 423 is responsible for the reconstruction of abscisic acid (ABA) signal transduction components in protoplasts, participates in ABA signal transduction and interacts directly with protein kinase (i.e., SnRK2.6) and ABA responsive element binding factor (ABF1) [44]. 2-methoxy-6-polyprenyl-1,4-benzoquinol methylase is involved in the synthesis of ubiquinone, which is also one of the characteristics of embryogenic callus. The above-mentioned proteins are potential embryonic markers, and they can more accurately reflect the internal mechanism of embryogenicity, although their determination is costlier than POD. The exact function of these proteins in *P. thunbergii* EVO technology needs to be verified in future studies.

## 4. Materials and Methods

### 4.1. Explant Treatment

Seeds of *P. thunbergii* were collected from 10 superior individuals of the excellent resistant provenances of Kunyushan in Yantai City (China). Seeds were kept at 4 °C for three weeks before being grown into seedlings. After disinfection (using 3% CuSO_4_ for 2 h), seeds were soaked in distilled water for 24 h and expanded seeds were distributed uniformly on filter paper in Petri plates. More than 3 cm of hypocotyls had grown after 10 d, and more than 1 cm of needles had grown after 30 d. The hypocotyls and needles were used as explants. The explants were sterilized with 75% alcohol for 30 s, washed with sterile water 3 times, sterilized with 2% NaClO for 10 min, and then washed with sterile water 4 times.

### 4.2. Effect of Different Factors on Callus Induction

The hypocotyls and needles were cut into 5–7 mm segments and inoculated to different media in a vertical-flow clean bench (Xin Yi Instrument and Meters Co., Ltd., Shanghai, China) to induce callus, then incubated in the dark at 23 ± 2 °C. A three-factor and four-level orthogonal experiment (a total of 16 combinations) was used to screen the best protocol for hypocotyls (Table 1). The three factors were medium type, 2,4-D concentration and 6-BA concentration. The four base media were woody plant medium (WPM), Murashige and Skoog medium (MS), B_5_ and DCR, with 2,4-D (1.0, 1.5, 2.0 and 2.5 mg L^−1^) and 6-BA (0.5, 1.0, 1.5 and 2.0 mg L^−1^) (Jinan Yalong Biotechnology Co., Ltd., Jinan, China). Each combination was examined with 200 biological replicates (20 Petri plates, 10 explants per Petri plate). In addition, 0.3 g L^−1^ PVP (Wuxi Yatai United Chemical Co., Ltd., Wuxi, China), 0.5 g L^−1^ CH (Beijing Hongrun Baoshun Technology Co., Ltd., Beijing, China) (adding by vacuum filtration), 0.5 g L^−1^ L-glutamine, 1.0 g L^−1^ inositol, 6.0 g L^−1^ agar and 20.0 g L^−1^ sucrose (Jinan Yalong Biotechnology Co., Ltd., Jinan, China) were supplemented to media. The growth rate and state of the callus were observed, and the induction rate was calculated. On the basis of the above experiments, the best medium type and hormone concentration ratio were found. These calli were subcultured using this preferred combination, subcultured once every 2 weeks, and then placed in differentiation medium (DCR + 10 mg L^−1^ ABA + 0.3 g L^−1^ PVP + 0.4 g L^−1^ gel + 0.5 g L^−1^ CH + 0.5 g L^−1^ L-glutamine + 0.5 g L^−1^ active carbon + 1 g L^−1^ inositol + 6 g L^−1^ agar + 10 g L^−1^ sucrose) for differentiation after 4 subcultures.

### 4.3. Microscope and Electron Microscope Observation

The calli at the induction stage, after two and four subcultures, and at the differentiation stage, were sampled, and 10 calli of EC and NEC were taken at each stage. The surface characteristics of calli were observed under a stereomicroscope (Carl Zeiss AG, Oberkochen, Germany) and scanning electron microscope (HITACHI, Tokyo, Japan). Some calli were stained with acetate magenta and Evans blue, then cytological observation was carried out under a biological microscope (Leica, Wetzlar, Germany). The goal of those observations was to identify whether they were EC [45]. The induction rate of EC could be calculated according to the proportion of EC to the total number of calli.

### 4.4. Determination of Antioxidant Enzyme Activity

According to the method of Kaewubon et al. [46], POD, SOD and catalase (CAT) activity in the callus samples were determined spectrophotometrically at 470, 560 and 240 nm measuring wavelength, respectively. For POD determination, samples, distilled water and three reagents in the kit (Solarbio Science & Technology Co., Ltd., Beijing, China) were added in turn to the determination tube, immediately mixed and timed. The POD activity was calculated using Equation (1):

(1) $$POD\;activity\;(Ug^{-1})=\frac{(A_{2}-A_{1})\times V_{1}\times V_{3}}{W\times V_{2}\times 0.01\times T}$$

where *A*_1_ is the absorbance value after 30 s, *A*_2_ is the absorbance value after 90 s, *V*_1_ is the total volume of the reaction system (1.07 mL), *V*_2_ is the volume of sample added (0.015 mL), *V*_3_ is the volume of extract added (1 mL), *W* is the weight of the sample (g), and *T* is the reaction time (1 min).

For SOD determination, the inhibiting percentage and SOD activity of each sample were calculated using Equations (2) and (3):

(2) $$Inhibiting\;percentage=\frac{\left({∆A}_{blank}-{∆A}_{determination}\right)}{{∆A}_{blank}}\times 100\%$$

where Δ*A_determination_* = *A_determination_* − *A_control_*, Δ*A_blank_* = *A*_1*blank*_ − *A*_2*blank*_.

(3) $$SOD\;activity\;(Ug^{-1})=\frac{inhibiting\;percentage\times V_{1}\times V_{3}}{(1-inhibiting\;percentage)\times W\times V_{2}}$$

where *V*_1_, *V*_2_, *V*_3_ and *W* have the same meaning as the above POD equation.

For CAT determination, the CAT activity was calculated according to Formula (4):

(4) $$CAT\;(Ug^{-1})=\frac{\Delta A\;\times \;V_{1}\;\times \;V_{3}\;\times \;T}{\varepsilon \;\times \;d\;\times \;10^{6}\;\times \;W\;\times \;V_{2}}=\frac{678\;\times \;\Delta A}{W}$$

where *V*_1_: total volume of reaction system, 1.035 × 10^6^ mL in this study; *V*_2_: volume of sample added, 0.035 mL; *V*_3_: volume of extraction solution added, 1 mL; *T*: reaction time, 1 min; *W*: the weight of sample, g; ε: molar absorbance coefficient of H_2_O_2_, 43.6 L/mol/cm in this study; d: optical diameter of cuvette, 1 cm; and Δ*A* = *A*_2_ − *A*_1_. *A*_1_ and *A*_2_: the initial absorbance and the absorbance after reaction.

### 4.5. Determination of SS Content

According to Vale et al. [47], the SS content in the callus samples was determined spectrophotometrically at 620 nm measuring wavelength and calculated using Equation (5):

(5) $$SS\;content\;(mg\;{mL}^{-1})=\frac{x\times V}{W}$$

where *V*: the total volume of sample, 10 mL; *W*: the weight of sample, g; and x: the concentration of sample, mg mL^−1^.

### 4.6. Determination of Quantitative Proteomics

Using EC and NEC as samples (each treatment having 3 samples, and each sample containing 3–5 calli), the label-free method was used to compare the proteomics of them. This method mainly included protein extraction, reduction and alkylation, peptide digestion and liquid chromatography–tandem mass spectrometry (LC-MS/MS) data acquisition. In the last step, the samples were separated by chromatography and analyzed by Q-Exactive mass spectrometer (Thermo Fisher, Waltham, MA, USA). The analysis time was 60 min, the detection mode was positive ion, the scanning range of the parent ion was 300–1800 *m*/*z*, the resolution of primary mass spectrometry was 70,000 at 200 *m*/*z*, AGC (automatic gain control) target was 3 × 10^6^, and maximum IT was 10 ms. The dynamic exclusion time was 40.0 s. The mass-to-charge ratios of peptides and peptide fragments were calculated according to the following methods: 10 MS2 scans were collected after each full scan. The MS2 Activation Type was HCD, the isolation window was 2 *m*/*z*, the resolution of MS2 was 17,500 at 200 *m*/*z*, normalized collision energy was 30 eV, and underfill ratio was 0.1%. The original mass spectrometry test file (Raw File) was used to retrieve the corresponding database with Maxquant software (v1.5.5.1), and finally the results of protein identification and quantitative analysis were obtained. Differentially expressed proteins (DEPs) were screened according to the standard of more than 2-fold change in expression (up-regulated more than 2-fold or down-regulated less than 0.5-fold).

Gene Ontology (GO) functional annotation was used to evaluate the significance level of protein enrichment of a GO functional item by Fisher’s Exact Test. The functional enrichment analysis of DEPs was based on the results of GO functional annotation, and all DEPs were compared with all proteins of the reference species or all proteins identified in this study. The significance of the difference between the EC and NEC was obtained by Fisher’s Exact Test, so as to find the functional categories of all DEP enrichment (*p* < 0.05).

### 4.7. Data Processing

An orthogonal design was used in this study, and IBM SPSS statistics (version 26) and Excel software (version 2019) were used for analysis of extreme difference (R), variance and interaction. Analysis of variance (ANOVA) for the orthogonal experiment was used to estimate the influence of each factor (medium type, 2,4-D concentration and 6-BA concentration), the influence of the interaction between the factors and determine which factor makes a significant difference (*p* < 0.05). For the factor with a significant effect, post hoc multiple comparison was performed using the LSD method to understand the significant difference between different levels of this factor. Significant differences among the treatments were detected by Duncan’s test (*p* < 0.05).

## 5. Conclusions

For *P. thunbergii* hypocotyl, the best protocol for EC induction was DCR + 2.5 mg L^−1^ 2,4-D + 1.0 mg L^−1^ 6-BA + 0.3 g L^−1^ PVP + 0.5 g L^−1^ CH + 0.5 g L^−1^ L-glutamine + 1.0 g L^−1^ inositol + 6.0 g L^−1^ agar + 20.0 g L^−1^ sucrose. Among the three influencing factors, only 2,4-D concentration had a significant effect on EC induction rate (*p* < 0.001), while the combination of 2,4-D and 6-BA had a significant interaction effect (*p* < 0.001). EC can be judged by morphology, higher POD, SOD and SS. Label-free quantitative proteomics analysis showed that PODs in EC were significantly up-regulated, and they were involved in antioxidant biological processes, located in the intercellular region, and performed molecular functions such as heme binding, so the POD index was the best physiological marker for EC. In addition, four up-regulated proteins and five EC-specific proteins were also found to be potential molecular markers. Among these proteins, glutathione S-transferase is beneficial to prevent cell death in EC, while indole-3-acetate O-methyltransferase 1-like and cytokinin dehydrogenase are beneficial to promote EC redifferentiation.

## Figures and Tables

**Table 1:** The induction rate of EC from hypocotyls through orthogonal experiment.

Number	Culture Medium Types	2,4-D Concentration/(mg L^−1^)	6-BA Concentration/(mg L^−1^)	Average Induction Rate (%)	Coefficient of Variation
1	WPM	1.5	2.0	82.1 ± 2.84	0.0346
2	WPM	2.0	1.0	79.2 ± 5.43	0.0686
3	WPM	1.0	0.5	49.7 ± 9.05	0.1821
4	WPM	2.5	1.5	81.4 ± 3.74	0.0460
5	MS	1.5	1.0	85.3 ± 3.59	0.0421
6	MS	2.0	2.0	83.9 ± 6.01	0.0716
7	MS	1.0	1.5	61.5 ± 10.49	0.1706
8	MS	2.5	0.5	76.3 ± 5.74	0.0752
9	B5	1.5	0.5	77.0 ± 2.52	0.0327
10	B5	2.0	1.5	77.9 ± 4.71	0.0605
11	B5	1.0	2.0	49.7 ± 5.20	0.1046
12	B5	2.5	1.0	86.8 ± 2.37	0.0273
13	DCR	1.5	1.5	79.8 ± 1.47	0.0184
14	DCR	2.0	0.5	85.2 ± 1.59	0.0187
15	DCR	1.0	1.0	56.0 ± 3.62	0.0646
16	DCR	2.5	2.0	88.6 ± 2.03	0.0229
k1	73.1	81.1	76.1		
k2	76.7	81.5	76.8		
k3	72.8	54.2	72.1		
k4	77.4	83.3	75.2		
R	4.6	29.0	4.8		

The k-value is the average of the factors at each level, and the optimal level of each factor can be judged by the k-value to derive the optimal combination of the test. R is the difference between the maximum and minimum values of the factors at each level, and a factor with a large R-value means that it has a large impact on the test result and is usually the main factor.

**Table 2:** Analysis of variance and interaction of influencing factors.

Factors	Sum of Squares of Deviations	Degree of Freedom	Mean Square	F	*p*	F_crit_
A	0.007	3	0.002	0.949	0.474	-
B	0.232	3	0.077	32.255	0.000 **	-
C	0.005	3	0.002	0.735	0.568	-
A × B	0.061	3	0.020	0.071	0.974	3.239
A × C	0.643	3	0.214	0.755	0.535	3.239
B × C	0.993	3	0.331	47.285	0.000 **	3.239
A × B × C	1.132	6	0.189	0.985	0.457	2.508

R^2^ = 0.944; **: *p* < 0.01; A, B and C represented medium type, 2,4-D concentration and 6-BA concentration, respectively.

**Table 3:** Post hoc multiple comparisons of 2,4-D concentrations.

	Level I	Level J	Average Value of Level I	Average Value of Level J	Difference Value (I − J)	*p*
**2,4-D concentrations**	1	2	0.811	0.815	−0.005	0.883
	1	3	0.811	0.542	0.268	0.000 **
	1	4	0.811	0.833	−0.022	0.516
	2	3	0.815	0.542	0.273	0.000 **
	2	4	0.815	0.833	−0.017	0.613
	3	4	0.542	0.833	−0.290	0.000 **

**: *p* < 0.01. The meanings of numbers 1–4 represent 1.5, 2.0, 1.0 and 2.5 mg L^−1^ respectively.

**Table 4:** Antioxidant enzyme activity and SS content between EC and NEC.

Samples	The POD Vitality (U g^−1^ FW)	The SOD Vitality (U g^−1^ FW)	The CAT Vitality (U g^−1^ FW)	SS Content (mg g^−1^ FW)
EC	36,166.00	106.85	511.77	80.08
	29,032.67	109.39	503.84	82.00
	36,736.67	100.75	586.94	91.32
Mean value	33,978.45 ± 4292.67 a	105.66 ± 4.44 a	534.18 ± 45.86 a	84.47 ± 6.01 a
NEC	15,363.38	50.19	434.08	30.23
	27,283.73	54.91	316.94	43.61
	19,506.57	53.70	658.49	40.00
Mean value	20,717.89 ± 6051.78 b	52.93 ± 2.45 b	469.83 ± 173.56 a	37.95 ± 6.92 b

Each index of each sample was measured three times. Different lowercase letters indicate a significant difference between EC and NEC (*p* < 0.05).

**Table 5:** Annotated proteins of up-regulated or specifically expressed proteins in EC.

Type	Function of Proteins	Main Protein Name
Up-regulated proteins	MF: FMN binding	Probable NAD(P)H dehydrogenase (quinone) FQR1-like 1, Probable NAD(P)H dehydrogenase (quinone) FQR1-like 2
	Heme binding	Cytochrome b5-like, **Peroxidase 4**, Flavonoid 3′-monooxygenase CYP75B3-like, **Cationic peroxidase 1**, Cytochrome P450 86A1, Cytochrome P450 98A1, Trans-cinnamate 4-monooxygenase
	Glutathione transferase activity	Glutathione S-transferase GSTU1, Probable glutathione S-transferase GSTU6, protein IN2-1 homolog B-like,
	BP: Response to oxidative stress	**Peroxidase 4**, **Cationic peroxidase 1**, **Phospholipid hydroperoxide glutathione peroxidase**, Ornithine aminotransferase
	Flavonoid biosynthetic process	Chalcone flavanone isomerase, Flavonoid 3′-monooxygenase CYP75B137, Chalcone synthase 1-like, Pinosylvin synthase
	Response to chemical	Probable glutathione S-transferase GSTU1, Probable glutathione S-transferase GSTU6, Protein IN2-1 homolog B-like
	CC: Cytosol	S-adenosylmethionine synthase 1, S-adenosylmethionine synthase 3, Cytosolic invertase 1, Glutathione S-transferase U20, Phosphoenolpyruvate carboxykinase, ATP-citrate synthase beta chain protein 1, Acetyl-CoA carboxylase 1, Diphosphomevalonate decarboxylase 2
	Extracellular region	**Peroxidase 4**, Endoglucanase 3-like, **Cationic peroxidase 1**, Nod factor hydrolase protein 1
	Cytoplasm	Isopentenyl-diphosphate Delta-isomerase II, Ribonuclease TUDOR 1, Phenylalanine ammonia-lyase, YTH domain-containing protein ECT4, Pyrophosphate-fructose 6-phosphate 1-phosphotransferase subunit beta, Probable inositol oxygenase, Probable aldo-keto reductase 2, Probable histone-arginine methyltransferase CARM1, Ornithine aminotransferase, Glutathione S-transferase U6, Oxygen-dependent coproporphyrinogen-III oxidase, **Superoxide dismutase [Cu-Zn]2**
Specifically expressed proteins	Related to cell division, auxin, cytokinin, abscisic acid and ubiquinone synthesis	Sister chromatid cohesion protein PDS5 homolog A, Cell division control protein 48 homolog E, Probable indole-3-acetic acid-amido synthetase GH3.1, indole-3-acetate O-methyltransferase 1-like, Tetraspanin-8, Cytokinin dehydrogenase, MLP-like protein 423, 2-methoxy-6-polyprenyl-1,4-benzoquinol methylase

Note: Annotated proteins are proteins with gene ID in NCBI (https://www.ncbi.nlm.nih.gov/). The up-regulated DEPs come from the top three items in MF, BP and CC of GO enrichment and the *p*-values are all less than 0.001. The proteins shown in the bold font are different types of POD and SOD.

## Data Availability

The datasets generated and/or analyzed during the current study are available from the corresponding author on reasonable request.
